# Machine learning-based approach for predicting low birth weight

**DOI:** 10.1186/s12884-023-06128-w

**Published:** 2023-11-20

**Authors:** Amene Ranjbar, Farideh Montazeri, Mohammadsadegh Vahidi Farashah, Vahid Mehrnoush, Fatemeh Darsareh, Nasibeh Roozbeh

**Affiliations:** 1 Fertility and Infertility Research Center, Hormozgan University of Medical Sciences, Bandar Abbas, Iran; 2 Mother and Child Welfare Research Center, Hormozgan University of Medical Sciences, Bandar Abbas, Iran; 3 Amirkabir University of Technology, Tehran, Iran

**Keywords:** Low birth weight, Fetal weight, Birth weight, Machine learning, X gradient boost model

## Abstract

**Background:**

Low birth weight (LBW) has been linked to infant mortality. Predicting LBW is a valuable preventative tool and predictor of newborn health risks. The current study employed a machine learning model to predict LBW.

**Methods:**

This study implemented predictive LBW models based on the data obtained from the “Iranian Maternal and Neonatal Network (IMaN Net)” from January 2020 to January 2022. Women with singleton pregnancies above the gestational age of 24 weeks were included. Exclusion criteria included multiple pregnancies and fetal anomalies. A predictive model was built using eight statistical learning models (logistic regression, decision tree classification, random forest classification, deep learning feedforward, extreme gradient boost model, light gradient boost model, support vector machine, and permutation feature classification with k-nearest neighbors). Expert opinion and prior observational cohorts were used to select candidate LBW predictors for all models. The area under the receiver operating characteristic curve (AUROC), accuracy, precision, recall, and F1 score were measured to evaluate their diagnostic performance.

**Results:**

We found 1280 women with a recorded LBW out of 8853 deliveries, for a frequency of 14.5%. Deep learning (AUROC: 0.86), random forest classification (AUROC: 0.79), and extreme gradient boost classification (AUROC: 0.79) all have higher AUROC and perform better than others. When the other performance parameters of the models mentioned above with higher AUROC were compared, the extreme gradient boost model was the best model to predict LBW with an accuracy of 0.79, precision of 0.87, recall of 0.69, and F1 score of 0.77. According to the feature importance rank, gestational age and prior history of LBW were the top critical predictors.

**Conclusions:**

Although this study found that the extreme gradient boost model performed well in predicting LBW, more research is needed to make a better conclusion on the performance of ML models in predicting LBW.

## Background

Birth weights less than 2500 g are called low birth weight (LBW). LBW has been linked to infant mortality and its consequences [1]. Predicting LBW is thus a valuable preventative tool and predictor of newborn health risks. Previous research has found that maternal demographics, preexisting health conditions, and prenatal care level are all linked to LBW [2, 3]. Thus, pinpointing which pregnant patients are most likely to have a baby with LBW during the preconception or early pregnancy stages is critical for saving neonatal lives and reducing potentially avoidable medical costs through direct clinical and health policy interventions. There are some documented studies on using ML in perinatal care and maternal health. Previous LBW prediction studies achieved good performance in predicting LBW; however, all previous studies recommended more studies due to study limitations such as small sample size or limited feature selection [4–7]. In this study, we aimed to evaluate the performance of eight different ML algorithms in predicting LBW.

## Methods

The findings of this retrospective cohort study are based on birth records obtained from the “Iranian Maternal and Neonatal Network (IMaN Net),” a legitimate national system, from January 2020 to January 2022. IMaN Net is a comprehensive system for registering maternal and newborn information on the outcomes of each delivery, which is completed daily by midwives in all birth centers and hospitals throughout Iran in an integrated manner. All patients’ personal information was deidentified and not disclosed.

Women with singleton pregnancies above the gestational age of 24 weeks who gave birth during a study period were included. The target population in this study was divided into LBW (≤ 2499 g) and not LBW (≥ 2500 g), which is the national standard definition [8]. Exclusion criteria included multiple pregnancies and fetal anomalies.

A predictive model was built using eight statistical learning models, including logistic regression, decision tree classification, random forest classification, deep learning feedforward, extreme gradient boost classification (XGBoost), light gradient boost (LGB), support vector machine (SVM), and permutation feature classification with k-nearest neighbors (KNN). Expert opinion and prior observational cohorts were used to select candidate LBW predictors for all models [9, 10]. Predictor factors included maternal age, educational level, maternal occupation, place of residence, inadequate prenatal care (less than three prenatal care visits), smoking, drug addiction, maternal anemia, cardiovascular disease, chronic hypertension, hepatitis, COVID-19, overt diabetes, gestational diabetes and thyroid dysfunction, parity, preeclampsia, fetal gender, method of childbirth, previous history of LBW, supplementary and vitamins intake were obtained from patient medical records. We used Chi-square test to evaluate the association between predicting factors mentioned above and LBW. Then we performed ML analysis approach. We followed the Guidelines for Developing and Reporting Machine Learning Predictive Models in Biomedical Research: A Multidisciplinary View to report our findings. The programming language Python was chosen to create the machine learning model. Scikit-learn was used to implement the ML algorithm. Scikit-learn is a machine-learning library written in Python. It includes an extensive collection of cutting-edge machine-learning algorithms for both supervised (including the multi-output classification and regression algorithm) and unsupervised problems [11].

Internal validation was carried out with the help of k-fold cross-validation. The cases were randomly assigned to either the “training set” (70%) or the “test set” (30%) using a random number generator. The original dataset kept the rate of LBW and non-LBW groups in the training and test sets constant. Using the training set, we arranged the parameters of the prediction models and evaluated their performance using the “test set”. The average performance was calculated by repeating these ten times.

Metrics, including area under the receiver operating characteristic curve (AUROC), accuracy, precision, recall, and F1 score, were used to assess the predictive power of the models. The accuracy metric calculates how often a model is correctly predicted across the entire dataset. Precision measures how many of the model’s “positive” predictions were correct. The model’s recall estimates how many positive class samples in the dataset were correctly identified. The F1 score combines precision and recall by using their harmonic mean, and maximizing the F1 score implies maximizing both precision and recall simultaneously. As a result, researchers have chosen the F1 score to evaluate their models in conjunction with accuracy. We used AUROC as the primary performance metric because it is a widely used index to describe the ML model’s ability to predict outcomes. The metrics were scaled from 0 to 1, with higher values indicating a better model [12].

## Results

Of 8850 eligible cases, we found 1280 women with a recorded LBW, for a frequency of 14.5%. The demographic and clinical characteristics of study population is given in Table 1. As it shown, maternal age, living residency, gestational age, parity, access to prenatal care, maternal anemia, chronic hypertension, preeclampsia, drug addiction, COVID-19, previous LBW, and newborn gender was linked to LBW.

**Table 1:** Demographic and clinical factors associated with low birth weight

Outcome	Non-LBW (*n* = 7570)	LBW (*n* = 1280)	*P*-value
**Maternal age**			< 0.001
13–19	137 (1.8)	36 (2.8)	
20–35	6247 (82.5)	995 (77.7)	
Above 35	1186 (15.7)	249 (19.5)	
**Education**			0.348
Illiterate	480 (6.3)	73 (5.7)	
Primary	2344 (31.0)	378 (29.5)	
High-school/Diploma	3463 (45.8)	609 (47.6)	
Advanced	1283 (16.9)	220 (17.2)	
**Occupation**			0.299
Housewife	6798 (89.8)	1146 (89.5)	
Worker/employee	772 (10.2)	134 (10.5)	
**Living residency**			0.045
Urban	5056 (66.8)	823 (64.3)	
Rural	2517 (33.2)	457 (35.7)	
**Gestational age**			< 0.001
24–36^+6^	429 (5.7)	801 (62.6)	
37–41	7141 (94.3)	479 (37.4)	
**Parity**			< 0.001
Primiparous	2056 (27.1)	439 (34.3)	
Multiparous	5517 (72.9)	841 (65.7)	
**Access to prenatal care**			0.030
Yes	7343 (97.0)	1255 (98.0)	
No	230 (3.0)	25 (0.2)	
**Maternal anemia**			0.047
No	7364 (97.2)	1233 (96.3)	
Yes	209 (2.8)	47 (3.7)	
**Chronic hypertension**			0.005
No	7501 (99.0)	1256 (98.1)	
Yes	72 (1.0)	24 (1.9)	
**Cardiovascular disease**			0.803
No	7492 (98.9)	1267 (99.0)	
Yes	81 (1.1)	13 (1.0)	
**Diabetes**			0.276
No	6420 (84.8)	1094 (85.5)	
Yes	1153 (15.2)	186 (14.5)	
**Preeclampsia**			< 0.001
No	7196 (95.0)	1083 (84.6)	
Yes	377 (5.0)	197 (15.4)	
**Drug addiction**			< 0.001
No	7530 (99.4)	1251 (97.7)	
Yes	42 (0.6)	29 (2.3)	
**Previous low birth weight**			< 0.001
No	7479 (98.8)	1089 (85.1)	
Yes	94 (1.2)	191 (14.9)	
**COVID-19**			0.020
No	7465 (98.6)	1250 (97.7)	
Yes	108 (1.4)	30 (2.3)	
**Thyroid dysfunction**			0.999
No	6778 (89.5)	1146 (89.5)	
Yes	795 (10.5)	134 (10.5)	
**Hepatitis**			0.079
No	7543 (99.6)	1279 (99.1)	
Yes	30 (0.4)	1 (0.1)	
**Newborn gender**			< 0.001
Male	3942 (52.1)	599 (46.8)	
Female	3631 (47.9)	681 (53.2)	
**Supplementary intake**			0.078
No	4 (0.1)	5 (0.4)	
Yes	7569 (99.9)	1275 (99.6)	

Data are presented as n (%)

**Fig. 1:**
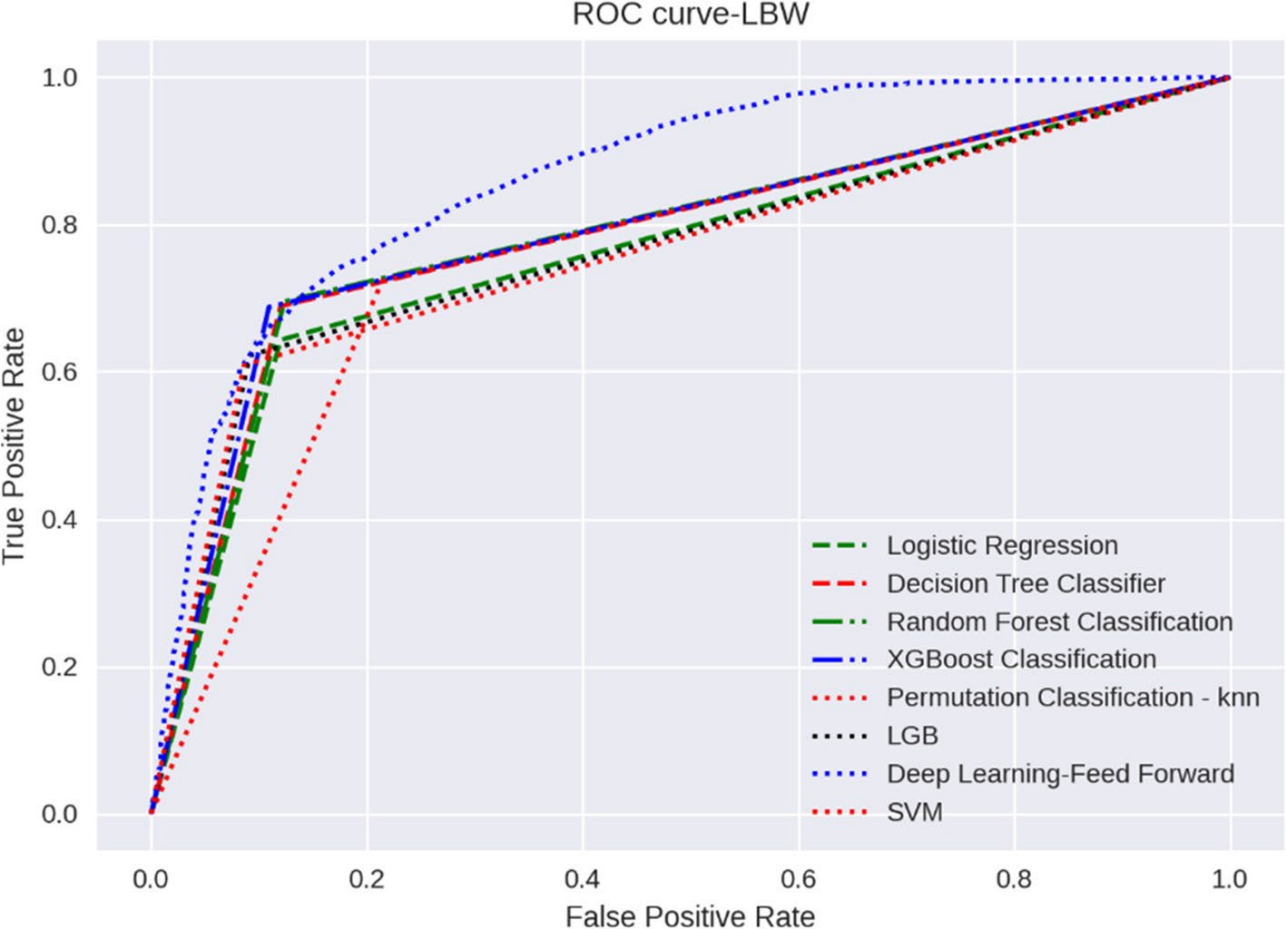
AUROC of ML models

In this study, we attempt to evaluate parameters and feature selection based on performance parameters using various ML algorithms. A plot ROC chart, as shown in Fig. 1, and calculate AUROC as a plot that allows the user to visualize the tradeoff between the classifier’s sensitivity. Deep learning (AUROC: 0.86), random forest classification (AUROC: 0.79), and XGBoost classification (AUROC: 0.79) all have higher ROC_AUC and perform better than others, as shown in Fig. 1.

Other performance parameters for each algorithm are shown in Table 2. Other performance parameters indicate that the XGBoost classification performs more than all. Random forest classification and deep learning feedforward are also very close. When the accuracy, precision, recall, and F1 score of the models mentioned above with higher AUROC were compared, the XGBoost model was the best model to predict LBW with an accuracy of 0.79, precision of 0.87, recall of 0.69, and F1 score of 0.77.

**Table 2:** Performance parameters of models with the highest AUROC

Row	Algorithms	Accuracy	Precision	Recall	F_1 Score
1	Random Forest Classification	0.78	0.85	0.70	0.77
2	XGBoost Classification	0.79	0.87	0.69	0.77
3	Deep Learning- Feed Forward	0.78	0.84	0.70	0.76

**Fig. 2:**
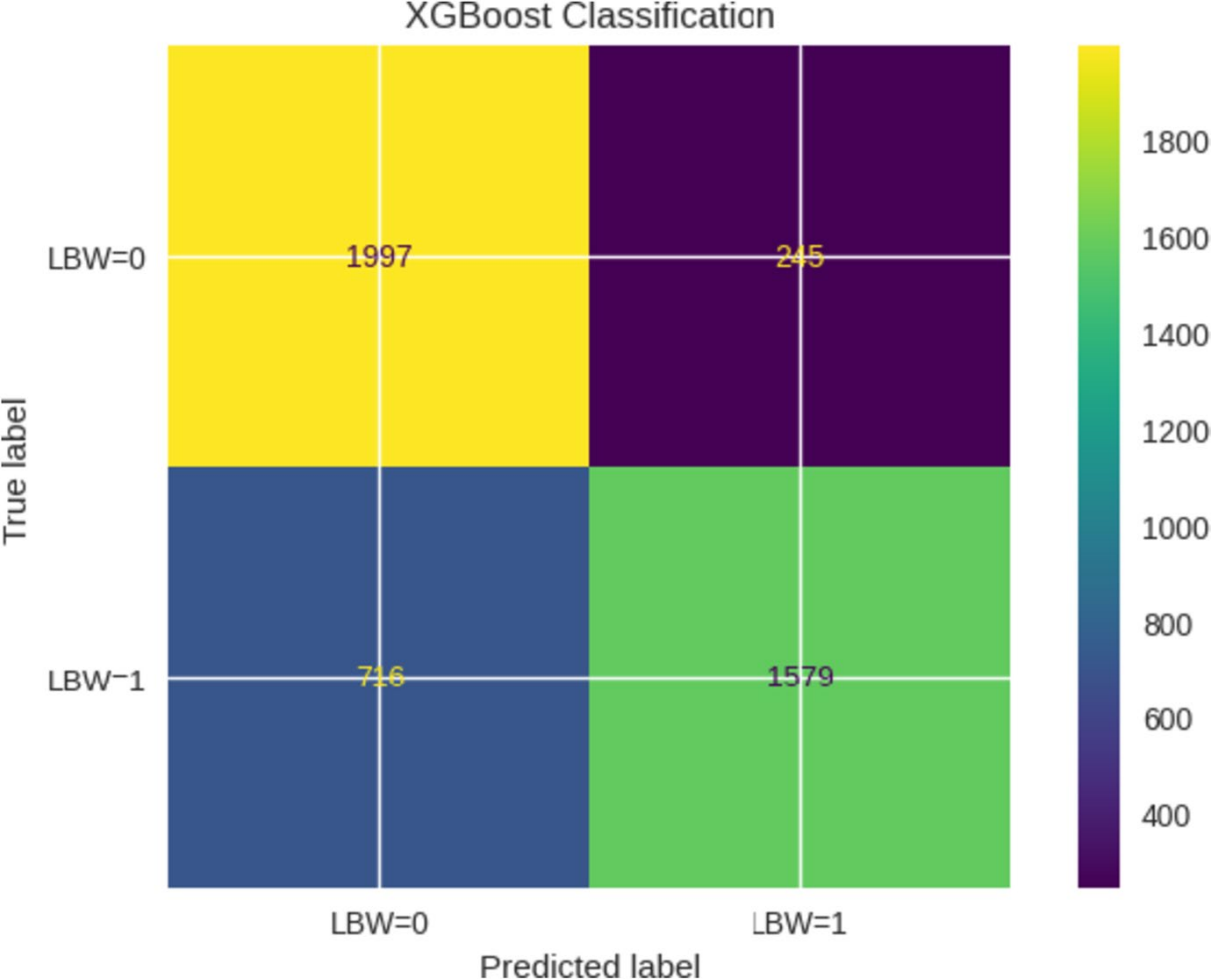
XGBoost classification confusion matrix

The confusion matrix of the XGBoost classification model is shown in Fig. 2.

**Fig. 3:**
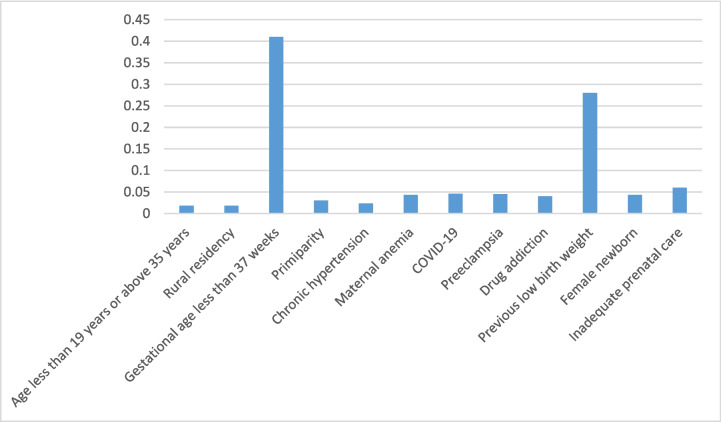
Feature importance of the XGBoost classification in the prediction of low birth weight

Figure 3 presents an analysis of the importance of variables in the XGBoost algorithm. As the feature importance rank was identified, gestational age and previous history of LBW were the top critical predictors.

## Discussion

With the exponential growth in the quantity and dimension of healthcare data in recent years, ML approaches for dealing with complex and high-dimensional data have been introduced [13–15]. In this study, we aimed to evaluate the performance of eight different ML algorithms in predicting LBW. According to our findings, the XGBoost classification model had a more significant diagnostic performance parameter with an AUROC of 0.79, accuracy of 0.79, precision of 0.87, recall of 0.69, and F1 score of 0.77. XGBoost classification is a supervised machine learning algorithm based on a distributed gradient-boosted decision tree [16]. It can produce consistent results while minimizing overfitting by employing a parallel tree-boosting strategy. Furthermore, XGBoost can use the importance score to determine the importance of each feature. Previous studies evaluating different ML machines for predicting LBW will also have promising results. According to Ahmadi et al., the random forest model performed well in terms of diagnostic performance, with an accuracy of 0.95, recall of 0.72, and AUROC of 0.89 [5]. Another study by Desiani et al. found that naive Bayes had excellent diagnostic performance, with an accuracy of 0.85 and a recall of 0.72 [17]. However, both studies were limited by a small sample size (less than 1000 participants).

Recent studies with larger sample sizes also demonstrated good performance. For example, in a survey by Eliyati et al., with a sample size of 12,500 study participants, SVM showed high diagnostic performance in predicting LBW with an accuracy of 0.93 [18]. Ren et al. used a more extensive study in this field, with a sample size of 266,687 birth records over six years. According to their findings, the XGBoost classification model had the highest recall score of 0.85, but the AUROC score was only 0.61 [19].

Although our study did not have the largest sample size of any study in this field, we believe that using hospital records made our feature selection rich enough to make a reasonable conclusion on identifying LBW risk factors. In our study, we surveyed maternal age, educational level, place of residence, inadequate prenatal care (fewer than three prenatal care visits), drug addiction, maternal anemia, cardiovascular disease, chronic hypertension, pyelonephritis, hepatitis, COVID-19, overt diabetes, gestational diabetes and thyroid dysfunction, parity, preeclampsia, and history of LBW. Among all the potential predisposing factors of LBW, gestational age and previous history of LBW were the top critical predictors. In line with previous findings [20, 21], gestational age is the highest predictor of LBW. Being born too soon (premature birth) is the most common cause of LBW. The prior history of LBW was another weighted factor in predicting LBW. It has been reported that the risk of LBW recurs between pregnancies. Women with a previous LBW baby have been identified as potential carriers of the recurrent risk and have a higher recurrence risk of LBW in their subsequent pregnancy than those with a previous normal birth weight baby [22]. Other factors, such as maternal comorbidities, sociodemographic characteristics, and fetal gender, were not among the weighted factors in predicting LBW, in contrast to previous studies. In one study, Bekele et al. found that fetal gender, marriage to birth interval, mother’s occupation, and mother’s age were all weighted factors in predicting LBW [23]. Another study found that maternal pre-pregnancy weight, mother’s age, number of doctor visits during the first trimester, and previous preterm labor were the most significant risk factors for LBW [4]. The differences in findings could be attributed to study design, the type of ML models used, geographical differences, or imbalances in each study’s dataset. It should be noted, however, that clinicians can use the key findings of each study to identify high-risk pregnant patients early in their pregnancy using early prenatal care screening tools.

Although we used a large dataset with a lot of maternal and neonatal information, a significant variable, like body mass index, was missing in most of the birth records, so we couldn’t use this factor in our selection features, which is a significant limitation of the study.

## Conclusion

Using ML approaches to predict LBW yielded promising results. As a result, this study might add to the current perinatal care literature. Although this study found that the XGBoost model performed well in predicting LBW, more research is needed to make a better conclusion on the performance of ML models in predicting LBW.

## Data Availability

The datasets generated and analyzed during the current study are available from the corresponding author upon reasonable request.

## Abbreviations

LBW: Low birth weight
ML: Machine learning
XGBoost: Extreme gradient boost
LGB: Light gradient boost
SVM: Support vector machine
KNN: K-nearest neighbors
